# Outcome of Surgery for Ischemic Mitral Regurgitation Depends on the Type and Timing of the Coronary Revascularization

**DOI:** 10.3390/jcm12093182

**Published:** 2023-04-28

**Authors:** Terézia B. Andrási, Alannah C. Glück, Olfa Ben Taieb, Ildar Talipov, Nunijiati Abudureheman, Lachezar Volevski, Ion Vasiloi

**Affiliations:** 1 Department of Cardiac Surgery, Philipps University of Marburg, 35043 Marburg, Germany; 2 School of Medicine, Philipps University of Marburg, 35032 Marburg, Germany; 3 Department of Cardiac Surgery, University of Basel, 4031 Basel, Switzerland

**Keywords:** ischemic mitral regurgitation, coronary revascularization, mitral valve surgery, survival

## Abstract

Objective: Long-term outcomes of mitral valve (MV) repair versus MV replacement for ischemic mitral regurgitation (IMR) in patients undergoing either prior (PCR) or concomitant coronary revascularization (CCR) by surgery (CABG) or intervention (PCI) are uncertain. Methods and Results: Of 446 patients receiving MV surgery for IMR between July 2006 and December 2010, 125 patients—87 CCR (69.1%) and 38 PCR (30.9%)—were eligible for inclusion in the study. Survival was higher in CCR versus PCR at long-term follow-up (78.83% vs. 57.9%, *p* = 0.016). The incidence of MACCE was lower in the CCR compared to PCR at both hospital discharge (34.11% vs. 63.57%, *p* = 0.003) and at follow-up (34.11% vs. 65.79%, *p* = 0.0008). Patients receiving CABG or CABG with PCI in PCR had higher mortality risks after MV surgery than CCR patients (X^2^ = 6.029, *p* = 0.014 and X^2^ = 6.466, *p* = 0.011, respectively). Whereas in the PCR group, MV repair and MV replacement achieved similar survival probability (X^2^ = 1.551, *p* = 0.213), MV repair in the CCR group led to improved survival compared to MV replacement (X^2^ = 3.921, *p* = 0.048). In MV replacement, LAD-CABG improved survival compared to LAD-PCI (U = 15,000.00, Z = −2.373 *p* = 0.018), and a substantial impact of arterial IMA-LAD grafting was revealed in the Cox-regression analysis (HR 0.334, CI: 0.113–0.989, *p* = 0.048) as opposed to venous-LAD grafting (HR 0.588, CI: 0.166–2.078, *p* = 0.410). Conclusion: Early treatment of IMR concomitant to coronary revascularization enhances long-term survival compared to delayed MV surgery after PCR. MV repair is not superior to MV replacement when performed late after coronary revascularization; however, MV repair leads to better survival than MV replacement when performed concomitantly with CABG with arterial LAD revascularization.

## 1. Introduction

Ischemic mitral valve regurgitation (IMR) caused by coronary artery disease is found in approximately 3% of all patients undergoing coronary angiography [1], and the presence of IMR is independently related to death after myocardial infarction [2].

Due to its inhomogeneous pathophysiology, IMR appears with all types of valve regurgitation as described in the Carpentier classification [3,4]. Therefore, the proper treatment of IMR is often debated, and the utility of revascularization with or without MV surgery is indeterminate [5].

Given the prevalence of IMR and the negative sequelae associated with chronic regurgitation [6,7,8], many surgeons consider the treatment of IMR at the time of CABG [9,10,11,12,13]. Although it has been questioned [11,12], milder IMR forms are treated solely with CABG [10], severe IMR caused by annular dilatation is treated with CABG and MV annuloplasty and the more complex forms of IMR are generally treated with CABG and valve MV replacement [4,5,12,13].

Whereas MV repair demonstrably provides better preservation of LV function [14] than MV replacement does without chordae-sparing procedures and produces superior freedom from valve-related complications [15] in patients with degenerative mitral insufficiency, similar advantages are still debated for IMR patients.

Moreover, recent studies [10,11,16] reveal that conservation or interventional treatment without addressing the IMR concomitantly with the treatment of coronary disease does not worsen long-term outcomes, and the benefits of surgical MV repair over replacement for IMR are controversial.

The aim of this study is to explore how the timing and the type of coronary revascularization influence the outcome after MV surgery for IMR, thus potentially aiding in surgical decision-making.

More precisely, the goal of this study was to determine whether survival was better after MV surgery with concomitant coronary revascularization (CCR) or when performed at a secondary point in time after previous coronary revascularization (PCR) in order to discover whether the type of MV surgery has an impact on survival as well as how the characteristics of coronary revascularization influence outcome after MV repair and replacement.

## 2. Methods

### 2.1. Study Population and Definition of IMR

This study was approved by the institutional review Board at the Philipps University of Marburg, including a waiver of informed consent (ek_mr_110221_Wensauer-1).

All patients who underwent mitral valve surgery from January 2010 to December 2017 were evaluated for inclusion (*n* = 467).

**Figure 1:**
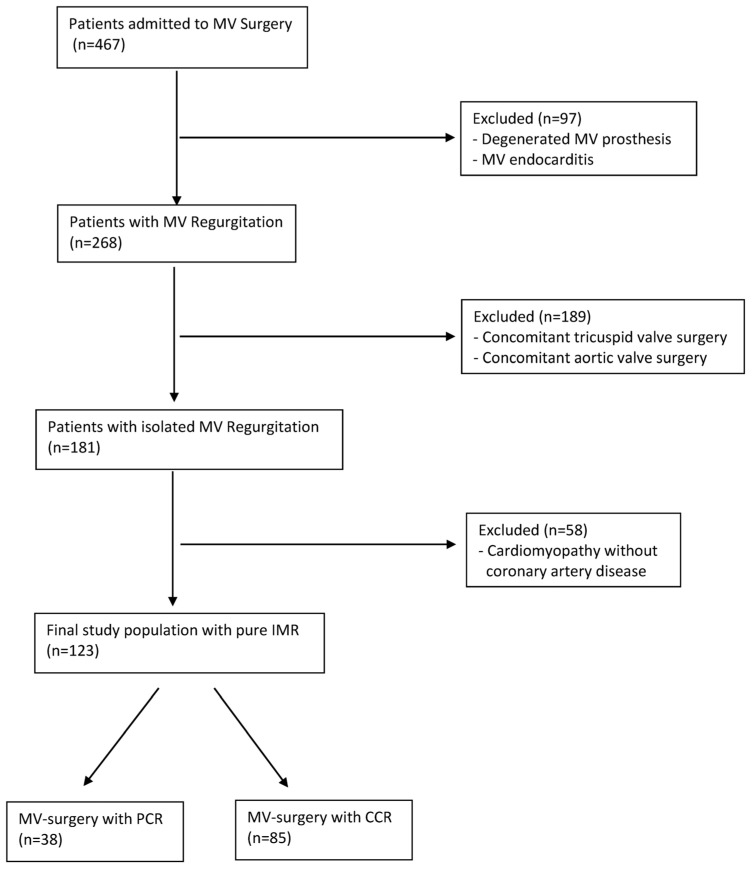
Flow chart.

Patients with a solely degenerative etiology, MV prosthesis, and/or MV endocarditis (*n* = 97), patients receiving concomitant tricuspid valve and/or aortic valve surgery (*n* = 189) and patients without coronary artery disease (*n* = 58) were excluded (Figure 1).

Of the remaining 367 patients, 243 suffered from coronary artery disease. Patients who had surgery with concomitant additional cardiac valve, aortic replacement, aortic root replacement, or atrial fibrillation-related procedures were excluded.

Of the remaining 123 patients, 85 received coronary revascularization concomitantly with MV surgery (CCR), whereas 38 patients received coronary revascularization at an earlier point in time (PCR). Mitral regurgitation was judged to be ischemic in origin when the valve leaflets and chordae were normal, and the regurgitation was caused by the consequences of myocardial infarction [17,18]. All patients in this study suffered from myocardial ischemia requiring coronary revascularization.

### 2.2. Data Collection and Variable Selection

Relevant history, preoperative, and postoperative study variables were obtained from clinical records. The extent of coronary disease was obtained from coronary angiograms. The type and timing of coronary revascularization were obtained from the interventional and surgical records. Echocardiography was performed in all patients to assess left ventricular function and quantify the degree of MV regurgitation according to the recommendations of the ESC Guidelines [19]. The operative strategies for percutaneous coronary intervention (PCI) and CABG were left to the discretion of the attending physician and patient wishes. The decision to perform MV repair or replacement was at the discretion of the attending surgeon. Early and late outcomes after MV surgery were collected.

Primary stratification was based on the timing of the coronary revascularization: prior coronary revascularization (PCR) versus concomitant coronary revascularization (CCR). Secondary stratification was based on the type of MV surgery: repair versus replacement. The third stratification was based on the type of coronary revascularization: CABG versus PCI.

### 2.3. Outcome Measures

The primary outcome measure was operative mortality and late mortality after MV surgery for IMR. Operative mortality was defined as 30-day or in-hospital death.

Survival of the IMR patients with PCR and CCR stratified by type of MV surgery (repair versus replacement) was evaluated as a secondary outcome measure.

The impact of the type of coronary revascularization on mortality after MV surgery was evaluated as a tertiary outcome measure.

### 2.4. Statistical Analysis

Categorical variables are presented as numbers with percentages and compared using Fisher’s exact test. Continuous variables are expressed as means with standard deviation and compared using two-sided *t*-tests or Mann–Whitney U tests.

Kaplan–Meier analysis was conducted to determine the probability of survival. Differences in survival rates between the PCR and CCR groups were examined by Mantel–Cox log-rank test. Similar analyses were conducted for patients undergoing MV repair and MV replacement.

The impact of the type of coronary revascularization on survival after MV repair and MV replacement was evaluated by logistic regression analysis and the Mann–Whitney U test.

## 3. Results

The study population consisted of 123 patients who met the inclusion criteria and underwent MV surgery for IMR, of whom 38 (30.9%) received PCR, and 85 (69.1%) received CCR.

### 3.1. Demographic Data

Baseline demographic and clinical characteristics are listed in Table 1. There were no differences between PCR and CCR in terms of age, clinical symptoms, laboratory data, and preoperative LV-EF.

Preoperative cardiac echography revealed a similar Carpentier classification of mitral pathology with predominant regurgitation Grade III-IV (89.47% and 83.29%, *p* = 0.428). Coronary angiography showed similar distribution and incidence of coronary pathology in the PCR and CCR groups with predominant triple-vessel disease (52.63% and 36.47%, *p* = 0.115), respectively.

### 3.2. Perioperative Outcome

Characteristics of the surgical treatment and postoperative outcome are presented in Table 2. The majority of patients (78.95% PCR and 72.94% CCR, *p* = 0.655) underwent elective surgery, with Redo-Sternotomy more often performed in PCR than in CCR (34.21% vs. 14.11%, *p* = 0.015).

The type of MV surgery did not differ between the groups, with 39.47% of PCR patients and 49.41% of CCR patients receiving MV repair (*p* = 0.334) and 34.21% and 31.76% receiving MV replacement with a biological prosthesis, respectively (*p* = 0.836).

Surgical coronary revascularization was more often performed in the CCR than in the PCR group (84.78% vs. 47.37%, *p* = 0.00003), with the LAD treated more often in the CCR than in the PCR (75.29% vs. 26.31%, *p* = 0.0005).

Postoperative outcome was similar in PCR and CRR patients in terms of reoperation rates (44.74% vs. 31.13%, *p* = 0.315) and length of hospital stay (22.34 ± 18.26 vs. 19.32 ± 13.76 days, *p* = 0.419).

The rate of MACCE at hospital discharge was significantly higher in the PCR group compared to CCR (*p* = 0.003), whereas the early mortality was similar between the two groups (*p* = 0.399).

Although the average follow-up occurred significantly later in the CCR compared to the PCR group (*p* = 0.016), mortality at follow-up was significantly lower in the CCR than in the PCR group (21.17% vs. 42.10%, *p* = 0.016). This difference seems to be the consequence of the more frequent non-cardiac causes of death encountered in PCR patients (Table 2).

### 3.3. Survival Outcomes

**Figure 2:**
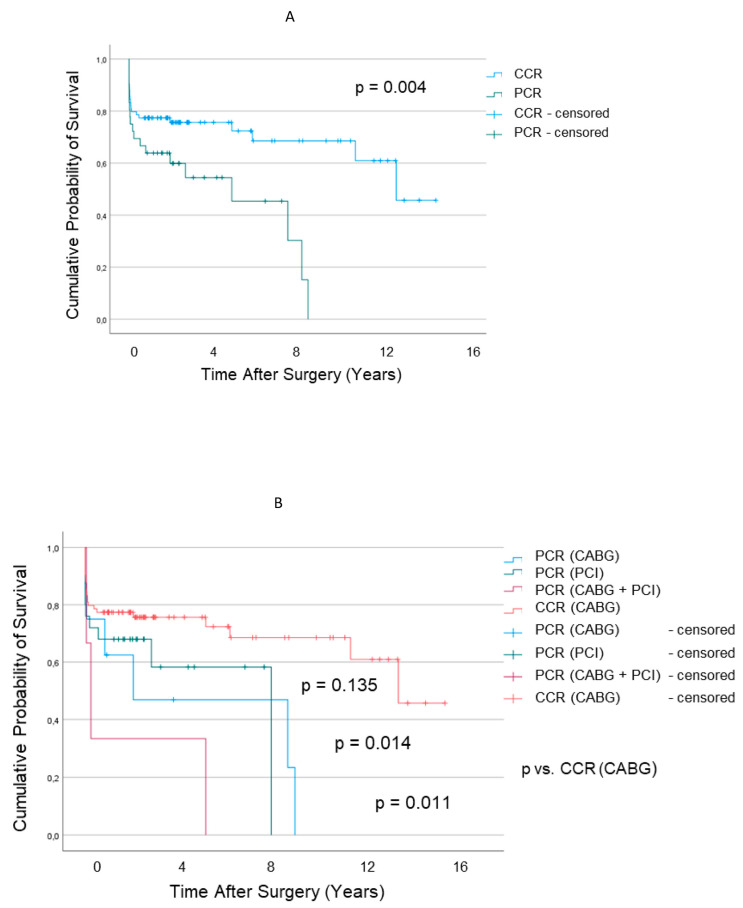
Estimated Survival after Mitral Valve Surgery for Ischemic Regurgitation in Patients with Concomitant (CCR) and Prior (PCR) Coronary Revascularization (**A**) and stratified based on CABG and PCI (**B**).

Survival at five years after MV surgery (Figure 2A) was significantly better in the CCR group than in PCR (*p* = 0.016).

Stratification by type of coronary revascularization (Figure 2B) revealed similar survival between PCR by PCI when compared to CCR (*p* = 0.013 and *p* = 0.148, respectively), whereas PCR by CABG and PCR by combined PCI + CABG were associated with significantly lower survival than CCR (*p* = 0.005).

**Figure 3:**
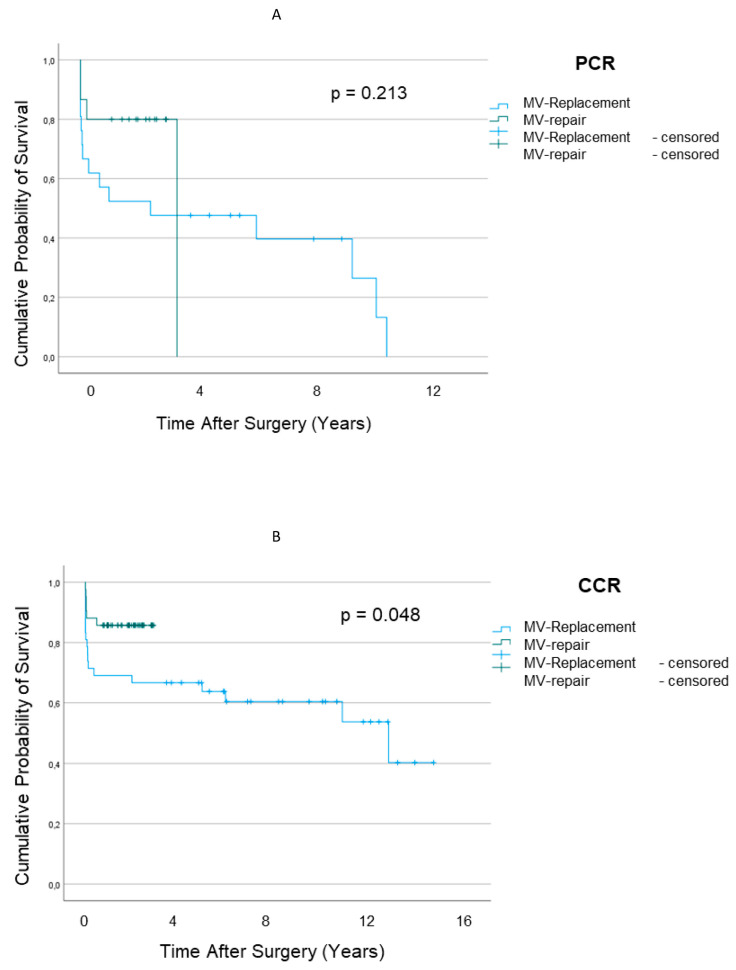
Estimated Survival after Mitral Valve Repair and Mitral Valve Replacement for Ischemic Regurgitation in Patients with PCR (**A**) and CCR (**B**) Coronary Revascularization.

Survival looking at any cardiac cause was similar between MR repair and MR replacement (*p* = 0.213) in the PCR patients (Figure 3A), whereas in CCR patients (Figure 3B), MV reconstruction yielded better survival than MR replacement (*p* = 0.048).

**Figure 4:**
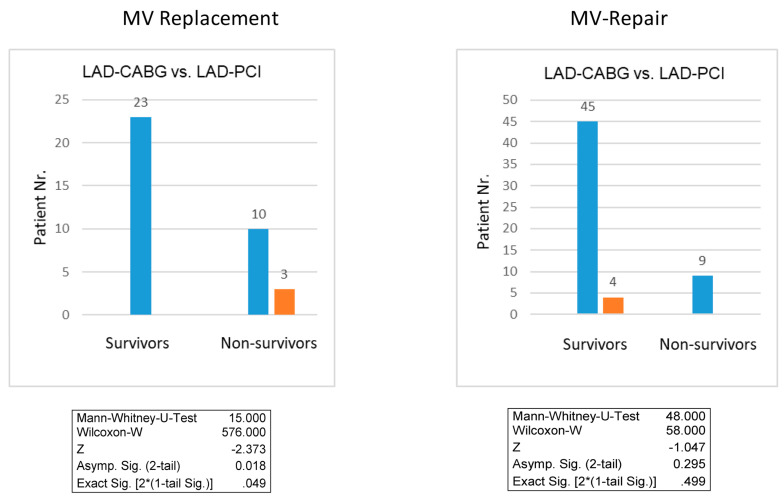
Impact of LAD Revascularization on Survival after MV replacement and MV repair.

The impact of the graft type on mortality after MV surgery with CCR (Table 3) revealed a significant role of arterial revascularization of the LAD in the MV replacement subgroup (*p* = 0.038), whereas no significant impact could be found in the MV repair subgroup (*p* = 0.916). This finding was confirmed by Mann–Whitney-U test and Wilcoxon-w test, revealing that surgical revascularization of the LAD is superior to PCI of the LAD when concomitant MV replacement is performed (*p* = 0.019 and *p* = 0.049, respectively, Figure 4A), whereas the type of LAD revascularization yields no difference in the MV repair subgroup (*p* = 0.295 and *p* = 0.499, respectively, Figure 4B).

## 4. Discussion

The results of the present study reaffirm that the type, as well as the timing of coronary revascularization, are important determinants of late survival after MV surgery for IMR. Three major findings are revealed: No. (1) MV surgery for IMR performed at the time as CABG (CCR) leads to a long-term survival benefit when compared to MV surgery performed late after coronary revascularization (PCR), especially when the prior revascularization involved surgical treatment; no. (2) MV repair shows no significant advantage over MV replacement when performed late after prior revascularization but is shown to improve long-term survival when performed concomitantly to CABG; and no. (3) grafting of the LAD with the internal mammary artery enhanced survival after MV replacement for IMR, without significant effect when performed concomitantly with MV repair.

According to many early publications on IMR [5,20,21], the role of MV surgery for moderate IMR at the time of CABG was initially de-emphasized since better outcomes were reported when mild ischemic mitral regurgitation was left untreated, and only coronary revascularization was performed [22,23]. The CTSN Moderate IMR trial randomized patients with moderate IMR to CABG alone or CABG + MV repair, with 2-year results demonstrating no improvement in survival, no differences in LV end-systolic volume index, and a higher early hazard of neurologic events in patients undergoing mitral valve repair [21]. Thus, initially, most patients with moderate IMR did not undergo MV intervention at the time of CABG [24], and these patients required MV surgery at a delayed point in time. Importantly, other randomized studies before the CTSN trial had demonstrated an improvement in left ventricular function with concomitant MV repair [25,26,27,28]. The presence of IMR was shown to be independently related to death after myocardial infarction [2]. Likewise, the more recent multicenter SAVE trial revealed that even a mild degree of MV regurgitation portended a substantial excess risk of cardiovascular mortality within 5 years after AMI [29], even in patients who did not have any overt signs of congestive heart failure at the time of the study. We believe that the differences in outcomes of these previous studies may be attributable to a higher proportion of viable myocardium and fewer previous myocardial infarctions in patients in the CTSN trial, in which case IMR may improve with revascularization alone without the need for MV surgery.

Trichon et al. [30] found a significant interaction between treatment strategy and the severity of CAD as measured by the Duke CAD index. CABG with or without MV surgery was associated with improved survival among patients with more severe CAD, while PCI was the preferred strategy among patients with less severe CAD. They also found no incremental advantage at five years after CABG plus MV surgery as opposed to CABG alone, regardless of CAD severity. The propensity score analysis revealed that CABG, as well as CABG plus MV surgery, had an improved survival rate over medical therapy.

The findings of our present study revealed that patients receiving MV surgery at a delayed stage after coronary revascularization (PCR group) had comparable survival to the CCR when early revascularization was performed by PCI, whereas patients who received CABG or CABG plus PCI had lower survival after MV surgery (Figure 2B). This distinction might be attributed to the variable degree of myocardial scarring associated with the different revascularization techniques and to the traumatic burden accompanying redo open surgery.

However, Miller [5] recognized that ignoring an important degree of IMR at the time of CABG is not prudent because it will only limit the potential functional benefit to be attained from the operation and compound the patient’s poor life expectancy. In agreement, our present results indicate that correction of IMR late after coronary revascularization does relatively little in terms of ameliorating the ravages of previous LV infarction or ischemia and leads to higher mortality than when performed concomitantly with coronary revascularization (Figure 2A).

Although MV repair demonstrably provides better preservation of LV function [14] than MV replacement without chordae-sparing procedures does and produces superior freedom from valve-related complications [15] in patients with degenerative MR, similar advantages could not be demonstrated for the PCR subset of our patients who were suffering from chronic IMR (Figure 3A).

Moreover, the amount of relative benefit of MV repair versus replacement in terms of survival was nearly erased in the PCR group, which is consistent with prior works describing that similar conclusions of MV surgical procedures were drawn whenever chronic LV wall motion abnormality was present [31].

Similarly, the CTSN severe IMR randomized controlled trial demonstrated that MV replacement for severe IMR resulted in a lower rate of recurrence of moderate or severe mitral regurgitation compared with MV repair along with lessened signs of heart failure and fewer cardiovascular readmissions; however, there was no difference in the number of adverse events or survival at two years post-treatment [32]. Mid-term survival was suboptimal but roughly equivalent between repair and replacement, also in the study by Calafiore et al. [33]. Gillinov [34] also concluded that patients receiving MV repair were not as sick as patients who received MV replacement.

Accordingly, our findings reveal that MV repair was not superior to replacement in the PCR group, probably because an improvement of LV contractility was not achievable late after ischemia and after delayed isolated MV surgery. Thus, our results confirm that chronic IMR is more of a LV disease than a valve disease.

Nonetheless, the efficacy of adding MV repair at the time of CABG is well demonstrated by Fattouch K et al. [25] and Chen et al. [26], who showed an improvement of the functional class of left ventricular ejection fraction and a decrease of regurgitation grade, left ventricular end-diastolic diameter, left ventricular end-systolic diameter, pulmonary artery pressure and left atrial size when compared with CABG alone. Moreover, CABG alone left more patients with heart failure symptoms at rest and during exercise. Although combined CABG and MV repair has no effect on survival at short-term follow-up, it was suggested that the positive trends that were evident were likely to become more significant with time (20,26,30).

Although current guidelines favor chordal-sparing mitral valve replacement over downsized annuloplasty repair for severe IMR (19, 21), the proportion of patients undergoing MV repair vs. MV replacement did not change over time in our patient cohort (PCR vs. CCR). This might have been the consequence of a row of publications similar to that of Cohn et al. [35] reporting that MV repair in patients with IMR was associated with a 5-times higher 5-year mortality than that seen after MV replacement.

In our study, MV repair was demonstrated to yield superior results to MV replacement in CCR patients where surgical coronary revascularization seems to be capable of saving the viability of a significant amount of myocardium (Figure 3B). This finding is consistent with the previous results of Kay et al. [36], demonstrating good results with MV repair for patients with IMR. In this regard, the CTSN trials [21,32] attributed the better results of repair to the higher rate of revascularization.

The negative effect of MV replacement on ventricular geometry and LV function can be reduced by preserving parts of the subvalvular apparatus of the MV [14,37].

Vassileva et al. [38] demonstrated in their meta-analysis that MV repair in patients with IMR improves short- and long-term survival compared to replacement when replacement is performed without preservation of the subvalvular apparatus. Importantly, the replacement patients were older and thus probably suffering from more chronic and extensive ischemic diseases. The myocardial scarring associated with the different revascularization techniques and the traumatic burden accompanying valve surgery was shown to increase the risk of sudden early cardiac arrest and mortality [38].

In agreement with these previous findings [37,39], our analysis reveals for the first time that substantial coronary revascularization has a significant impact on outcome after MV surgery for IMR (Table 3), with the type of revascularization playing a pivotal role. Whereas LAD-CABG is superior to LAD-PCI in patients receiving MV replacement, LAD-CABG, and LAD-PCI yield similar outcomes when performed in addition to MV repair (Figure 4).

Gillinov et al. [31] revealed by chance that arterial revascularization of the LAD significantly improves outcomes after MV surgery for IMR. Accordingly, our findings confirm surgical arterial LAD revascularization as a predictor for outcome after MV replacement, suggesting that viability and contractility of the left ventricle are of primordial importance for the postoperative outcome, especially when the integrity of the subvalvular apparatus during MV replacement cannot be achieved.

Finally, our data emphasize that detecting and quantifying IMR is essential for risk stratification before MV surgery.

## 5. Limitations

This study was limited by its observational nature. Given the retrospective nature, there is a selection bias due to a lack of explanation of why surgeons performed MV repair or MV replacement in some patients but not in others.

The small patient number in the PCR group did not permit further stratification of MV repair and replacement based on the type of coronary revascularization (CABG, PCI, and CABG with PCI).

## 6. Conclusions

IMR addressed at the time of coronary revascularization improves long-term outcomes when compared to MV surgery performed at a later point in time after coronary revascularization. Although PCI tends to lead to a better outcome than CABG in PCR patients, CABG, or the addition of CABG to PCI before MV surgery, significantly worsens the outcome.

Although MV repair is not superior to MV replacement when performed late after coronary revascularization, MV repair leads to better survival than MV replacement when performed concomitantly with CABG.

Surgical arterial revascularization of the LAD plays a significant role in improving long-term outcomes after MV replacement; however, it is not superior to PCI when associated with MV repair.

## 7. Future Research

Having the prerequisite of MV repair concomitant with CABG, our results might suggest that hybrid interventional therapy of IMR by PCI and Mitral-Clip implantation might be at least as good as full surgical treatment.

Such an alternative approach could be considered for IMR patients whenever PCI would be capable of recruiting and maintaining substantial myocardial viability of the left ventricle; however, additional research is required to prove this hypothesis.

## Figures and Tables

**Table 1:** Preoperative Patient Data.

	CCR			PCR			*p*-Value
Nr. Patients	87 (69.6%)			38 (30.4%)			
Demographics							
Age	67.69	±	10.31	69.94	±	8.68	0.244
BMI	26.73	±	3.87	27.97	±	4.05	0.047
Male Gender	31		(36.47)	12		(31.57)	0.685
Previous Cardiac Therapy							
CABG	0		(0.0)	10		(26.31)	0.0001
PCI	0		(0.0)	25		(65.78)	0.0001
CABG + PCI	0		(0.0)	3		(7.89)	0.028
Clinical Symptoms							
Angina Pectoris	17		(20.00)	3		(7.89)	0.179
Dyspnea	60		(70.59)	31		(81.58)	0.267
Acute Heart Failure	8		(9.41)	4		(10.52)	0.540
Laboratory Data							
Troponin (ng/l)	1614.81	±	4451.41	507	±	151.56	0.784
CK-MB (U/l)	62.05	±	69.65	35.28	±	60.60	0.813
Echocardiography							
Pre-OP LV-EF (%)	49.91	±	10.48	44.16	±	12.44	0.746
MR Grade I-II	14		(16.71)	4		(10.53)	0.428
MR Grade III-IV	71		(83.29)	34		(89.47)	0.428
Predominant Carpentier Type I	32		(37.65)	13		(34.21)	0.840
Predominant Carpentier Type II	34		(40.00)	14		(36.84)	0.842
Predominant Carpentier Type IIIa	16		(18.82)	7		(18.42)	1.000
Predominant Carpentier Type IIIb	2		(2.35)	3		(7.89)	0.322
Coronary Angiography							
Left Main-CAD	8		(9.41)	1		(2.63)	0.272
1-Vessel-CAD	29		(34.12)	10		(26.32)	0.530
2-Vessel-CAD	25		(29.41)	8		(21.05)	0.385
3-Vessel-CAD	31		(36.47)	20		(52.63)	0.115
LAD	55		(64.70)	15		(39.47)	0.011
D 1	12		(14.12)	3		(7.89)	0.389
D 2	1		(1.18)	0		(0.00)	1.000
IM	6		(7.06)	1		(2.63)	0.435
(L)CX	29		(34.12)	11		(28.95)	0.678
OM 1	8		(9.41)	1		(2.63)	0.272
OM 2	4		(4.71)	4		(10.53)	0.251
RCA	39		(45.88)	15		(39.47)	0.559
PDA	2		(2.35)	1		(2.63)	1.000
(R)AM	4		(4.71)	2		(5.26)	1.000

CCR, concomitant coronary revascularization; PCR, prior coronary revascularization; LV-EF, left ventricular ejection fraction; MR, mitral valve regurgitation; CAD, coronary artery disease.

**Table 2:** Present Surgical Treatment and Postoperative Outcome.

	CCR			PCR			*p*-Value
	87 (69.6)			38 (30.4)			
Elective	62		(72.94)	30		(78.95)	0.655
Urgent	15		(17.65)	6		(15.79)	1.000
Emergent	8		(9.41)	2		(5.26)	0.722
First Sternotomy and First CPB	73		(85.88)	25		(65.79)	0.015
Redo-Sternotomy and Re-CPB	12		(14.11)	13		(34.21)	0.015
MV repair	42		(48.3)	15		(39.47)	0.334
MV replacement	45		(51.7)	23		(60.52)	0.334
- Biological Prosthesis	28		(31.86)	13		(34.21)	0.836
- Mechanical Prosthesis	17		(19.84)	10		(26.32)	0.350
Coronary Revascularization (total)							
LAD System	64		(75.29)	10		(26.31)	0.0005
(L)CX System	8		(9.41)	1		(2.63)	0.277
RCA System	23		(27.05)	6		(15.78)	0.252
Coronary Surgery (CABG)	72		(84.78)	18		(47.37)	0.00003
- 1 Bypass	28		(32.94)	14		(36.84)	0.685
- 2 Bypasses	19		(21.18)	2		(5.26)	0.033
- 3 Bypasses	25		(29.41)	2		(5.26)	0.002
Coronary Intervention (PCI)	13		(15.29)	4		(10.53)	0.580
- 1-Stent	10		(11.76)	3		(7.89)	0.372
- 2-Stent	3		(3.53)	0		(0.00)	0.552
- 3-Stents	0		(0.00)	1		(2.63)	0.309
Post-OP MR I	23		(31.08)	8		(25)	0.654
Post-OP LV-EF (%)	47.59	±	10.78	46.61	±	9.64	0.553
Hospital Stay (days)	19.32	±	13.76	22.34	±	18.26	0.419
MACCE at Hospital Discharge	29		(34.11)	24		(63.57)	0.003
Mortality at Hospital Discharge	17		(19.54)	10		(26.32)	0.399
Follow-Up Time (years)	11.6	±	0.69	4.68	±	0.73	0.016
MACCE at Follow-up	29		(34.11)	25		(65.79)	0.0008
Mortality at Follow-up	24		(21.17)	19		(42.10)	0.016
Cardiac Causes of Death	15		(17.13)	9		(23.79)	0.452
Cardiogenic Shock	11		(12.6)	5		(13.2)	1.000
- Myocardial Infarction	2		(2.40)	2		(5.31)	0.580
Cardiac Bleeding	1		(1.20)	0		(0.0)	1.000
Cardiac Failure with MOF	1		(1.20)	2		(5.71)	0.225
Non-Cardiac Causes of Death	8		(9.41)	9		(23.68)	0.045
Septical Shock	5		(6.02)	6		(11.42)	0.456
Gastro-intestinal Bleeding	0		(0.0)	1		(2.86)	0.309
Pneumonia	1		(1.20)	0		(0.0)	1.000
Stroke	1		(1.20)	2		(5.71)	0.225

CCR, concomitant coronary revascularization, PCR, prior coronary revascularization MV, mitral valve; MR, mitral valve regurgitation.

**Table 3:** Effect of Type of Coronary Revascularization on Mortality after Mitral Valve Surgery for Ischemic Valve Disease.

		Univariable Analysis					Multivariable Analysis				
Covariate		Coeff.	OR	95% CI		*p*-Value	Coeff.	OR	95% CI		*p*-Value
MV replacement											
LAD	Arterial	−1.522	0.218	0.058	0.819	0.024	−0.883	0.413	0.179	0.954	0.038
	Venous	−1.606	0.201	0.038	1.062	0.059	−0.759	0.468	0.157	1.394	0.173
	PCI	−1.058	0.347	0.045	2.669	0.309	0.778	2.176	0.285	16.62	0.453
OM	Venous	2.898	18.14	1.639	20.818	0.018	−0.402	0.669	0.151	2.965	0.597
	PCI	−0.528	0.590	0.073	4.767	0.621	−0.007	0.993	0.134	7.331	0.994
RCA	Arterial	−14.594	0.000	0.000		0.984					
	Venous	0162	1.176	0.462	2.993	0.734					
	PCI	0.289	1.335	0.302	5.901	0.703					
PDA	Arterial	−0.615	0.541	0.000		0.984					
	Venous	0.874	2.396	0.302		0.997					
MV repair											
LAD	Arterial	0.730	2.076	0.504	8.553	0.312					
	PCI	0.778	2.176	0.285	16.623	0.453					
OM	Arterial	−13.434	1.728	0.000		0.050	−13.100	0.000	0.000	0.000	0.991
	Venous	−27.556	0.000	0.000			0.547	1.728	0.216	13.830	0.606
	PCI	1.296	3.656	0.000	1.296						
RCA	Arterial	−12.998	0.000	0.000		0.988					
	Venous	1.308	3.699	0.732	18.690	0.114					
	PCI	1.844	6.322	1.258	31.778	0.025	0.874	2.396	0.000	0.000	0.997
PDA	Arterial	−10.374	0.000	0.000		0.957					
	Venous	0.753	2.124	0.790	5.711	0.136					
	PCI	−0.652	0.521	0.000	0.000	0.998					

## Data Availability

The data underlying this article is available in the article.
